# Distinct Human NK Cell Phenotypes and Functional Responses to *Mycobacterium tuberculosis* in Adults From TB Endemic and Non-endemic Regions

**DOI:** 10.3389/fcimb.2020.00120

**Published:** 2020-03-24

**Authors:** Levelle D. Harris, Jeremiah Khayumbi, Joshua Ongalo, Loren E. Sasser, Joan Tonui, Angela Campbell, Felix Hayara Odhiambo, Samuel Gurrion Ouma, Galit Alter, Neel R. Gandhi, Cheryl L. Day

**Affiliations:** ^1^Emory Vaccine Center, Emory University, Atlanta, GA, United States; ^2^Center for Global Health Research, Kenya Medical Research Institute, Kisumu, Kenya; ^3^Department of Epidemiology, Rollins School of Public Health, Emory University, Atlanta, GA, United States; ^4^Ragon Institute of MGH, MIT, and Harvard, Cambridge, MA, United States; ^5^Division of Infectious Diseases, Department of Medicine, Emory University School of Medicine, Atlanta, GA, United States; ^6^Department of Microbiology and Immunology, Emory University School of Medicine, Atlanta, GA, United States

**Keywords:** *Mycobacterium tuberculosis*, LTBI, NK cells, innate immunity, phenotype

## Abstract

*Mycobacterium tuberculosis* (Mtb) is the causative agent of tuberculosis (TB), which leads to an estimated 1. 5 million deaths worldwide each year. Although the immune correlates of protection against Mtb infection and TB disease have not been well-defined, natural killer (NK) cells are increasingly recognized as a key component of the innate immune response to Mtb and as a link between innate and adaptive immunity. In this study, we evaluated NK cell phenotypic and functional profiles in QuantiFERON-TB (QFT)^+^ and QFT^−^ adults in a TB endemic setting in Kisumu, Kenya, and compared their NK cell responses to those of Mtb-naïve healthy adult controls in the U.S. We used flow cytometry to define the phenotypic profile of NK cells and identified distinct CD56^dim^ NK cell phenotypes that differentiated the Kenyan and U.S. groups. Additionally, among Kenyan participants, NK cells from QFT^+^ individuals with latent Mtb infection (LTBI) were characterized by significant downregulation of the natural cytotoxicity receptor NKp46 and the inhibitory receptor TIGIT, compared with QFT^−^ individuals. Moreover, the distinct CD56^dim^ phenotypic profiles in Kenyan individuals correlated with dampened NK cell responses to tumor cells and diminished activation, degranulation, and cytokine production following stimulation with Mtb antigens, compared with Mtb-naïve U.S. healthy adult controls. Taken together, these data provide evidence that the phenotypic and functional profiles of NK cells are modified in TB endemic settings and will inform future studies aimed at defining NK cell-mediated immune correlates that may be protective against acquisition of Mtb infection and progression to TB disease.

## Introduction

Infection with *Mycobacterium tuberculosis* (Mtb) can lead to development of active tuberculosis (TB) disease, which is currently the leading cause of death in the world due to a single infectious agent (WHO Publication, 2018a). The vast majority of individuals infected with Mtb remain asymptomatic and are considered to have latent Mtb infection (LTBI). Approximately one quarter of the global population is estimated to harbor Mtb infection (Houben and Dodd, 2016), with 10 million individuals developing active TB disease each year (WHO Publication, 2018a). The only currently licensed TB vaccine, *Mycobacterium bovis* bacille Calmette-Guérin (BCG), provides variable efficacy, ranging from 0 to 80%, against pulmonary TB disease in adults (Andersen and Scriba, 2019).

Both innate and adaptive immunity, including Mtb-specific T cell and antibody (Ab) responses, are clearly important in maintaining control of Mtb (Lu et al., 2016; Simmons et al., 2018), although the precise immune correlates of protection to Mtb infection have not been well-defined. Natural killer (NK) cells are increasingly recognized as a key component of the innate immune response to Mtb and as a link between innate and adaptive immunity (Gabrielli et al., 2016; Choreno Parra et al., 2017). IFN-γ production by NK cells activates antimicrobial effector functions of macrophages, which is essential for control of Mtb; furthermore, secretion of granulysin by NK cells can kill intracellular Mtb when delivered by perforin (Stenger et al., 1998; Lu et al., 2014). Studies of Mtb infection in T cell-deficient mice indicated that NK cell-mediated IFN-γ production contributes significantly to inhibition of bacterial replication (Feng et al., 2006). Mtb can bind directly to TLR2 on NK cells (Esin et al., 2013) as well as the natural cytotoxicity receptor (NCR) NKp44 (Esin et al., 2008). Human NK cells can lyse Mtb-infected macrophages *in vitro* via interactions with c-type lectins and NCRs expressed on NK cells (Korbel et al., 2008), and suppress growth of Mtb in infected monocytes (Yoneda and Ellner, 1998; Brill et al., 2001). IL-22 production by NK cells inhibits intracellular growth of Mtb *in vitro* by enhancing phagolysosomal fusion (Dhiman et al., 2009). NK cells are recruited to the lung in patients with active TB disease (Portevin et al., 2012). However, NK cells circulating in peripheral blood of patients with pulmonary TB disease exhibit decreased IFN-γ production capacity (Bozzano et al., 2009; Garand et al., 2018), which is partially restored following anti-TB treatment (Nirmala et al., 2001), thus suggesting an association between NK cell functional capacity and bacterial load. Moreover, longitudinal cohort studies have indicated that progression to active TB disease is preceded by a decline in the frequency of circulating NK cells, which is restored following successful treatment for active TB (Roy Chowdhury et al., 2018), thus providing further evidence of an important role for NK cells in Mtb infection and TB disease in humans.

NK cell activity is tightly regulated through a sophisticated network of numerous germline-encoded activating and inhibitory receptors (Bryceson et al., 2006), the variegated expression of which generates heterogenous populations of NK cells with high diversity (Horowitz et al., 2013; Strauss-Albee et al., 2014). Moreover, NK cell surface marker expression changes in the settings of inflammation, infection and cancer, and increasing evidence indicates NK cells can differentiate into distinct subsets with specialized functions, referred to as “adaptive” NK cells (Tesi et al., 2016; Freud et al., 2017). Adaptive NK cells in humans have been defined most clearly in the context of human cytomegalovirus (HCMV) infection, which has been associated with expansion of distinct NK cell subsets and enhanced responsiveness to virally-infected cells in an antibody-dependent manner (Wu et al., 2013; Zhang et al., 2013; Lee et al., 2015; Schlums et al., 2015). Antigen-specific NK cells have also been described in simian immunodeficiency virus (SIV)-infected and vaccinated rhesus macaques (Reeves et al., 2015). In humans, infection with HCMV and other viruses leads to expansion of NK cell subsets with adaptive features expressing CD57 and the activating receptor NKG2C (Guma et al., 2004; Bjorkstrom et al., 2011; Lopez-Verges et al., 2011; Beziat et al., 2013). Adaptive NK cells in HCMV infection are characterized by downregulation of receptors such as NKp30 and NKp46 (Guma et al., 2004), downregulation of the transcription factors PLZF and IKZF2 and loss of intracellular adaptor signaling molecules (Lee et al., 2015; Schlums et al., 2015). Downregulation of these molecules has been associated with pronounced changes in DNA methylation patterns (Lee et al., 2015; Schlums et al., 2015), thus clearly demonstrating pathogen-induced epigenetic reprogramming as a mechanism driving the generation of adaptive NK cells.

In addition to HCMV, NK cells with adaptive features have been described in other chronic human infections, including human immunodeficiency virus (HIV), hepatitis C virus (HCV), and Epstein-Barr virus (EBV) (Paust et al., 2017). The potential for persistent bacterial infections, such as Mtb, to promote adaptive diversification of NK cell is less clear. In a mouse model of TB, vaccination with BCG induces memory-like NK cells producing IFN-γ, which provide protection against challenge with Mtb (Venkatasubramanian et al., 2017). In humans, recent studies in South Africa indicate that BCG revaccination of individuals with LTBI boosts BCG-reactive NK cell responses for at least 1 year after revaccination (Suliman et al., 2016). NK cells from patients with active TB disease exhibit decreased expression of the activating NCRs NKp30 and NKp46 (Bozzano et al., 2009), a phenotype consistent with changes in adaptive NK cell phenotypic profiles in HCMV infection (Guma et al., 2004).

While increasing evidence from animal models and humans indicates that NK cells can differentiate into distinct subsets with specialized functions, it currently remains unclear if Mtb exposure and infection modifies NK cell phenotypic and functional signatures, and if so, how Mtb-associated changes in the NK cell repertoire may impact acquisition of Mtb infection and/or progression to active TB disease. To test the hypothesis that Mtb exposure and infection are associated with distinct NK cell phenotypic and functional profiles, we recruited a cohort of HIV-negative, Mtb-infected and uninfected adults in a TB endemic setting in Kisumu, Kenya and a cohort of Mtb-naïve, healthy adults in the U.S., a non-TB endemic setting. We performed a comprehensive analysis of NK cells from individuals in these cohorts and identified distinct CD56^dim^ NK cell phenotypic profiles that differentiated Kenyan adults from U.S. adults. Moreover, we demonstrated that CD56^dim^ phenotypic profiles correlated with dampened NK cell responses to MHC class I-devoid cells and diminished reactivity to Mtb antigen stimulation in Kenyan adults.

## Materials and Methods

### Study Participants

Kenya Cohort: Blood samples were collected from individuals ≥18 years of age enrolled at the Kenya Medical Research Institute Clinical Research Center in Kisumu, Kenya. Study participants included adults with a normal chest x-ray and no symptoms of TB disease and no previous history of diagnosis or treatment for active TB disease. Mtb infection status was evaluated by QuantiFERON®-TB Gold In-Tube (QFT; Qiagen). Individuals with a positive QFT result (TB Antigen-Nil ≥0.35 IU/ml) were defined as having LTBI. Individuals with a TB Antigen-Nil response <0.35 IU/ml were defined as healthy controls (QFT^−^). Serologic testing for HIV antibodies was done for all individuals using the Diagnostic Kit for HIV (1+2) Antibody V2 (*KHB*® Shanghai Kehua Bio-engineering Co., Ltd). All participants enrolled for the study were seronegative for HIV. HCMV seropositivity in healthy Kenyan adults is 97% (Njeru et al., 2009), thus participants enrolled in Kisumu are presumed to be HCMV seropositive. *U.S. healthy controls*: Blood samples were collected from healthy adults enrolled at the Emory Vaccine Center in Atlanta, GA. U.S. healthy adult controls were U.S.-born, had not been vaccinated with BCG, and had no history of exposure to TB. All U.S. healthy controls were seropositive for HCMV IgG antibodies, as measured using the Cytomegalovirus IgG ELISA kit (Abnova).

### Ethics Statement

This study was conducted in accordance with the principles expressed in the Declaration of Helsinki. All subjects provided written informed consent for participation in the study, which was approved by the Kenya Medical Research Institute Scientific and Ethics Review Unit and the Emory University Institutional Review Board.

### PBMC Isolation

Blood samples from all participants were collected in sodium heparin tubes for isolation of peripheral blood mononuclear cells (PBMCs). PBMCs were isolated via density gradient centrifugation, cryopreserved, and stored in LN_2_ until use. Cryopreserved PBMCs were thawed in a 37°C water bath and resuspended in 10 ml RPMI 1640 (Corning) with deoxyribonuclease I (DNase, 10 μg/ml, Sigma-Aldrich) and washed twice with RPMI 1640. Cells were then suspended in R10 (RPMI 1640 supplemented with 10% heat-inactivated fetal calf serum [FCS], 100 U/ml penicillin, 100 μg/ml streptomycin, and 2 mM L-glutamine) and used in phenotypic and functional NK cell assays described below.

### NK Cell Phenotyping

Thawed PBMCs were washed in PBS and stained with Zombie NIR™ Fixable Viability Dye (BioLegend) for 15 min at room temperature. Cells were washed with PBS and surface stained for 30 min in the dark at room temperature with anti-CD3 Alexa Fluor 700 (BioLegend; UCHT1), anti-CD14 Alexa Fluor 700 (BioLegend; HCD14), anti-CD19 Alexa Fluor 700 (BioLegend; HIB19), anti-CD56 Brilliant Violet (BV) 711 (BioLegend; HCD56), anti-CD16 BV 605 (BD; 3G8), anti-NKG2A PE (Beckman Coulter; IM329IU), anti-NKG2D BV 421 (BioLegend; 1D11), anti-NKp30 Alexa Fluor 647 (BioLegend; P30-15), anti-NKp46 PE-Cy7 (BioLegend; 9E2), and anti-CD57 FITC (BioLegend; HCD57). After incubation with conjugated antibodies, cells were washed with PBS and fixed with 2% paraformaldehyde (PFA).

A second phenotyping panel was designed to measure the NK cell expression of intracellular markers. PBMCs were stained with Zombie NIR™ Fixable Viability Dye and surface stained for 30 min at room temperature with anti-CD56 BV 711 (BioLegend; HCD56), anti-CD16 BV 605 (BD; 3G8), anti-CD3 Alexa Fluor 700 (BioLegend; UCHT1), anti-CD14 Alexa Fluor 700 (BioLegend; HCD14), anti-CD19 Alexa Fluor 700 (BioLegend; HIB19), and anti-TIGIT PE (BioLegend; A15153G). Cells were washed in PBS and suspended in FoxP3 Fixation Buffer (eBioscience) for 30 min on ice. After fixation, cells were washed with FoxP3 Permeabilization Buffer (eBioscience) and stained with anti-granzyme B PE-CF594 (BD; GB11), anti-perforin PE-Cy7 (BD; BD48), and anti-granulysin Alexa Fluor 488 (BD; RB1) for 30 min in the dark at room temperature. Finally, cells were washed with FoxP3 Staining Buffer and resuspended in PBS.

### Stimulation and Staining of NK Cells

NK cell responses to target cells were evaluated using K562 and p815 tumor cell lines (ATCC). Just prior to use in NK cell stimulation experiments, p815 cells were incubated with rabbit anti-mouse polyclonal lymphocyte serum (Cedarlane) for 30 min to coat the cells with Ab. Ab-coated p815 cells were then washed with R10 before being added to PBMCs. Donor PBMCs were incubated with K562 or Ab-coated p815 cells at an effector to target ratio of 10:1. PBMCs incubated in R10 media alone served as a negative control. CD107a PE-Cy7 (BioLegend; H4A3), brefeldin A (5 μg/ml; Sigma-Aldrich), and monensin (5 μg/ml; BioLegend) were added to each sample at the beginning of stimulation. Cells were incubated at 37°C degrees for 5 h.

NK cell responses to Mtb antigens were evaluated by stimulation of PBMCs with 10 μg/ml of *Mycobacterium tuberculosis* H37RV derived cell wall, cell membrane and whole cell lysate (obtained from BEI Resources, NIAID, NIH; catalog numbers NR14828, NR14831, and NR14822, respectively). PBMCs were incubated in R10 with anti-CD107a PE-Cy7 (BioLegend; H4A3), recombinant human IL-2 (100 U/ml; NIH AIDS Research and Reference Reagent Program, Catalog #136), and Mtb antigens for 24 h at 37°C and 5% CO_2_. Brefeldin A (5 μg/ml; Sigma-Aldrich) and monensin (5 μg/ml; BioLegend) were added at the final 5 h of incubation. PBMCs incubated in R10 media with anti-CD107a and IL-2 alone served as a negative control. For cytokine neutralization experiments, PBMCs were incubated with purified NA/LE mouse anti-human IL-12 (p40/p70) (BD; C8.6), purified mouse anti-human IL-18 (R&D Systems; 125-2H), or purified NA/LE mouse IgG1 κ isotype control (BD; 107.3) for 15 min prior to addition of Mtb antigens, as described above.

Following stimulation of PBMCs with either target cell lines or Mtb antigens, cells were washed with PBS and stained with Zombie NIR™ Fixable Viability Dye (BioLegend) for 15 min. Cells were washed with PBS and surface stained with anti-CD56 BV 711 (BioLegend; HCD56), anti-CD16 BV 605 (BD; 3G8), anti-CD3 Alexa Fluor 700 (BioLegend; UCHT1), anti-CD14 Alexa Fluor 700 (BioLegend; HCD14), anti-CD19 Alexa Fluor 700 (BioLegend; HIB19), anti-CD158a FITC (BioLegend; HP-MA4), anti-CD158b FITC (BioLegend; DX27), and anti-CD158e1 FITC (BioLegend; DX9) for 30 min at room temperature in the dark. Stained cells were washed with PBS and fixed with FoxP3 Fixation Buffer (eBioscience) for 30 min on ice. Cells were washed with FoxP3 Permeabilization Buffer (eBioscience) and stained intracellularly with anti-CD69 PerCP-Cy5.5 (BioLegend; FN50), anti-IFN-γ BV 480 (BD; B27), anti-TNFα Alexa Fluor 647 (BioLegend; MAB11), and anti-IL-22 PE (BioLegend; 2G12A41) for 30 min at room temperature in the dark. Cells were washed in PBS and prior to acquisition on a BD LSRII flow cytometer.

### Flow Cytometry and Data Analysis

Cells were acquired on a BD LSRII flow cytometer with BD FACSDiva software (v8.0) and analyzed with FlowJo software (v9.6; BD). Compensation was performed using single-stained anti-mouse Ig,κ beads (BD Bioscience). Single cells were identified by plotting forward scatter height and forward scatter area. Lymphocytes were identified by plotting forward scatter height and side scatter height. Viable lymphocytes were identified by low expression of NIR viability dye. NK cells were identified as live lymphocytes negative for CD3, CD14, CD19, and positive for the NK lineage markers CD56 and/or CD16. NK cells were further stratified based on intensity of the expression of CD56 (CD56^dim^, CD56^bright^, and CD56^neg^ NK cells).

### Data Analysis and Statistics

A minimum of 1,000 NK cells was acquired in each panel for each individual. Expression of functional markers by NK cells (CD69, IFN-γ, CD107a, TNF-α, and IL-22) were analyzed after subtraction of background expression in the negative control condition. A non-parametric Mann-Whitney test was used to compare differences between two groups. Differences between 3 groups were evaluated using a non-parametric Kruskal-Wallis test, with *p-*values adjusted for multiple comparisons using Dunn's post-test. Correlations were evaluated using Spearman's rank-order correlation. *P* < 0.05 were considered significant.

## Results

### Study Participants

Blood samples were collected from 61 participants in Kisumu, Kenya and from 9 Mtb-naïve healthy adult controls in Atlanta, GA (U.S.) (Table 1). Participants enrolled in Kenya were stratified by QFT result: QFT^+^ (considered to have LTBI, *n* = 31) and QFT^−^ (*n* = 30). The majority of participants from Kisumu were household contacts of an active TB patient within 2 years prior to study enrollment (20/31 QFT^+^ individuals [64.5%] and 21/30 QFT^−^ individuals [70%]). All three groups were similar with regard to age and sex characteristics, with the exception that U.S. healthy controls were older than Kenya QFT^−^ participants.

**Table 1:** Characteristics of study participants.

**Participant group**	***n***	**Age, y^a^ (IQR)**	**Sex** **(% male)**	**QFT, IFN-γ** **IU/ml^b^ (IQR)**
Kenya QFT^+^	31	32 (23–53)	26	8.84 (3.22–10.00)
Kenya QFT^−^	30	24 (21–33)	37	0.00 (0.00–0.05)
U.S. Healthy Controls	9	43 (33–60)^c^	22	N/A

a Value denotes median age in years.

b Value denotes median.

c p < 0.05, compared with Kenya QFT^−^.

*IQR, interquartile range*.

### NK Cells in QFT^+^ and QFT^−^ Kenyan Adults Are Characterized by Increased Proportions of CD56^neg^ NK Cells

**Figure 1:**
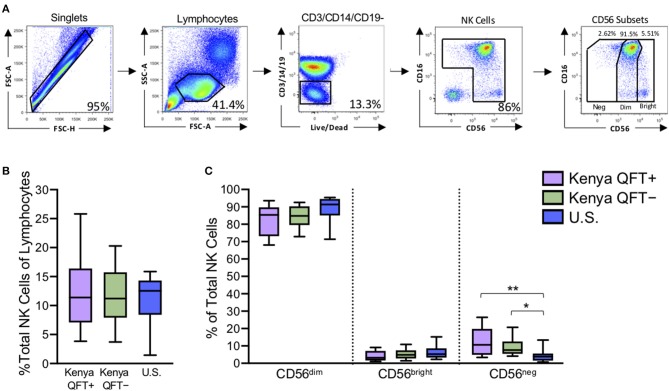
Kenyan adults exhibit higher proportions of CD56^neg^ NK cells, compared with U.S. adults. Flow cytometry was used to identify NK cell subsets in PBMCs from Kenyan adults (*n* = 31 QFT^+^; *n* = 30 QFT^−^) and U.S. adult healthy controls (U.S., *n* = 9). **(A)** Flow cytometry gating strategy for NK cells and CD56 subsets (CD56^neg^, CD56^dim^, CD56^bright^). **(B)** Frequency of total NK cells as a percentage of lymphocytes. **(C)** Frequency of CD56 subsets as a proportion of total NK cells. Boxes in **(B,C)** represent the median and interquartile ranges; whiskers represent the 10th and 90th percentiles. Differences among groups were assessed using a Kruskal-Wallis test, with *p*-values adjusted for multiple comparisons using Dunn's post-test. ^*^p < 0.05 and ^**^p < 0.01.

Redistribution of NK cell subsets, including expansion of the CD56^neg^ subset, has been described in the setting of chronic viral infections (Bjorkstrom et al., 2010). To determine if the frequency and distribution of NK cell subsets are modified in the setting of Mtb infection, we used flow cytometry to measure NK cells directly *ex vivo* in PBMCs from all three participant groups (Figure 1A). The frequency of total NK cells in the lymphocyte population was similar between the three participant groups (Figure 1B). As expected, CD56^dim^ NK cells constituted the dominant subset of NK cells in all participant groups (Figure 1C). There were no significant differences in the proportions of CD56 subsets between QFT^+^ and QFT^−^ Kenyan groups, thus indicating that Mtb infection does not significantly modify the frequency and distribution of NK cells subsets in peripheral blood. However, both QFT^+^ and QFT^−^ Kenyan adults had significantly higher proportions of CD56^neg^ NK cells, compared with Mtb-naïve healthy adult controls in the U.S. (Figure 1C).

### CD56^dim^ NK Cells Exhibit Distinct Phenotypic Profiles in Kenyan and U.S. Adults

**Figure 2:**
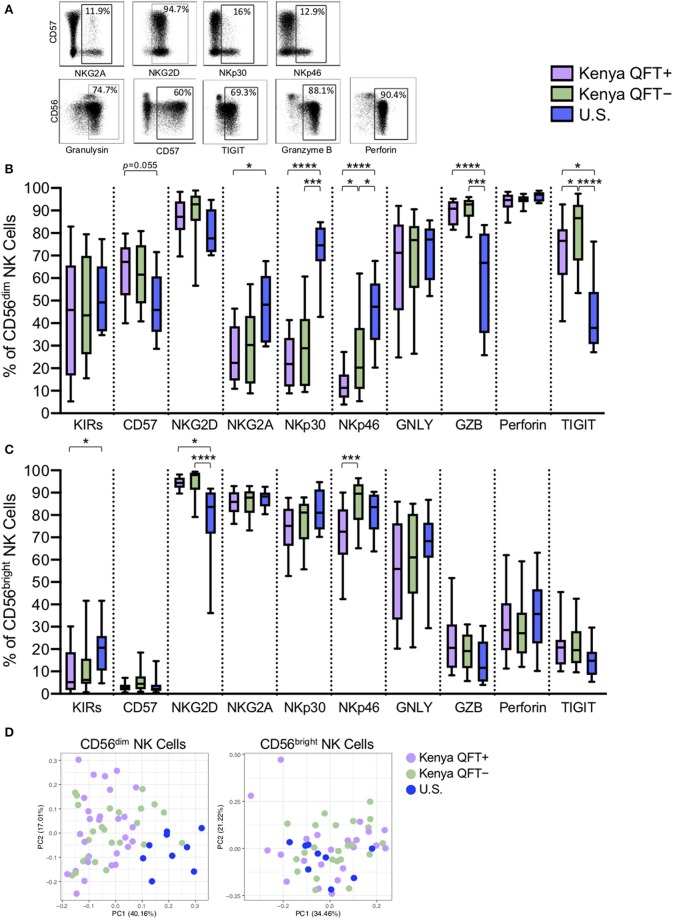
CD56^dim^ NK cells exhibit distinct phenotypes in Kenyan and U.S. adults. Flow cytometry was used to evaluate the phenotype of NK cells in PBMCs from each of three participant groups. **(A)** Representative flow plots from a Kenyan QFT^+^ individual demonstrating expression of the indicated phenotypic markers; plots are shown gated on total NK cells. **(B)** Frequency of CD56^dim^ NK cells expressing each phenotypic marker. **(C)** Frequency of CD56^bright^ NK cells expressing each phenotypic marker. GNLY, granulysin; GZB, granzyme B. **(D)** Principal component analysis (PCA) of expression of the 10 phenotypic markers on CD56^dim^ NK cells (left panel) and CD56^bright^ NK cells (right panel) from each of the three participant groups. Boxes in **(B,C)** represent the median and interquartile ranges; whiskers represent the 10th and 90th percentiles. Differences among groups in **(B,C)** were assessed using a Kruskal-Wallis test, with *p*-values adjusted for multiple comparisons using Dunn's post-test. ^*^p < 0.05; ^***^p < 0.001; and ^****^p < 0.0001.

Given that NK cell surface marker expression can change in the setting of infection (Freud et al., 2017), we performed flow cytometry to evaluate expression of 10 phenotypic markers expressed by NK cells in each of the three participant groups (Figure 2A). Since CD56 subsets have distinct transcriptional profiles (Collins et al., 2019), we determined the phenotypic profiles of CD56^dim^ and CD56^bright^ NK cells subsets separately. There was a progressive increase in expression of the differentiation marker CD57 by CD56^dim^ NK cells from U.S. healthy controls to QFT^−^ and QFT^+^ Kenyan individuals. By contrast, NKG2A, NKp30, and NKp46 were progressively decreased on CD56^dim^ NK cells from U.S. healthy controls to QFT^−^ and QFT^+^ Kenyan individuals (Figure 2B). While the cytotoxic molecules perforin and granulysin were expressed at similar levels among the three groups, expression of granzyme B was markedly increased in both groups of Kenyan participants, compared with U.S. healthy controls. Moreover, expression of the inhibitory receptor TIGIT was significantly higher on CD56^dim^ NK cells from Kenyan participants, compared with U.S. healthy controls. Similar phenotypic differences were also found when evaluating the phenotype of the total NK cell population in the three participant groups (Figure S1).

While there were substantial differences in the phenotypic profile of CD56^dim^ NK cells between Kenyan and U.S. adults, we also identified phenotypic markers that were expressed at significantly different levels between QFT^+^ and QFT^−^ Kenyan adults, with lower expression of both NKp46 and TIGIT on CD56^dim^ NK cells from QFT^+^ individuals, compared with QFT^−^ individuals (Figure 2B). These data indicate that human Mtb infection may be associated with downregulation of specific receptors expressed by circulating CD56^dim^ NK cells.

By contrast with CD56^dim^ NK cells, fewer differences were found among the three participant groups when evaluating the phenotypic profiles of CD56^bright^ NK cells. Although expression of NKG2D was similar among the groups on CD56^dim^ NK cells, NKG2D expression was significantly higher on CD56^bright^ NK cells from QFT^+^ and QFT^−^ Kenyan adults, compared with U.S. healthy controls (Figure 2C). Importantly, similar to CD56^dim^ cells, expression of NKp46 was also significantly lower on CD56^bright^ NK cells from Kenyan QFT^+^ individuals, compared with QFT^−^ individuals. PCA of expression of the 10 phenotypic markers by CD56^dim^ cells from the three groups indicated that U.S. healthy controls can be clearly differentiated from Kenyan individuals. By contrast, PCA of the same 10 phenotypic markers by CD56^bright^ NK cells in the same individuals does not clearly distinguish the participant groups (Figure 2D).

Taken together, these data indicate substantial differences in CD56^dim^ NK cell phenotypic profiles distinguish Kenyan and U.S. adults. Moreover, these data also indicate that among Kenyan adults residing in a TB endemic environment, expression levels of the natural cytotoxicity receptor NKp46 and the inhibitory receptor TIGIT are further modified in QFT^+^ adults with LTBI, compared with QFT^−^ adults.

### CD56^dim^ NK Cells From Kenyan Adults Have Dampened Responses to Tumor Cells, Compared With U.S. Adults

**Figure 3:**
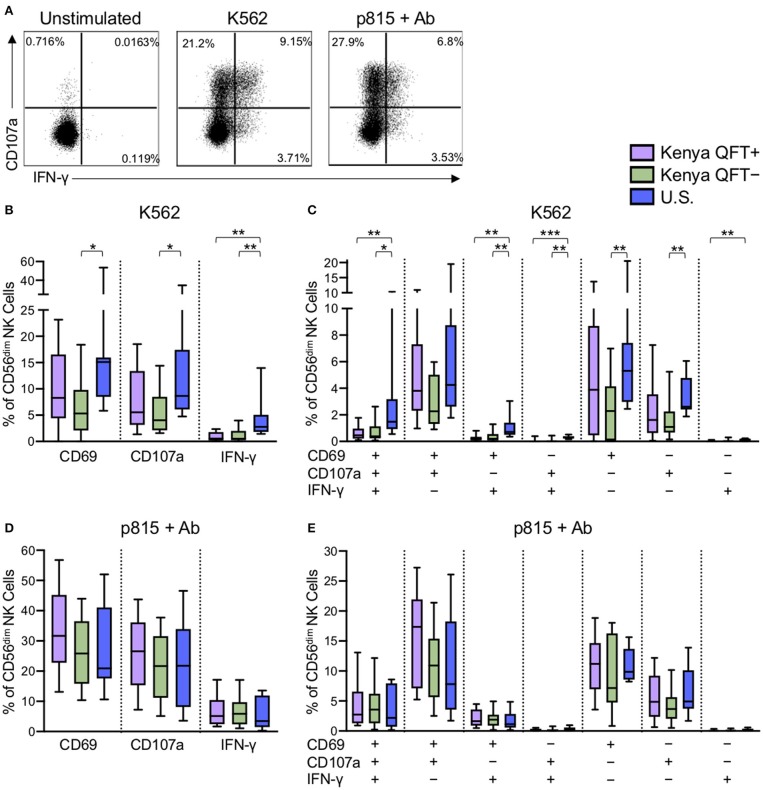
NK cells from Kenyan adults have dampened responses to MHC class I-devoid target cells, but similar ADCC responses, compared with U.S. adults. PBMCs were stimulated for 5 h with either MHC class I-devoid K562 cells or with Ab-coated p815 cells. NK cells were evaluated by flow cytometry for expression of CD69, CD107a, and IFN-γ. **(A)** Representative flow plots from a U.S. healthy control of CD107a and IFNγ production by CD56^dim^ NK cells following stimulation with K562 cells or Ab-coated p815 cells. **(B,D)** Expression of CD69, CD107a and IFN-γ by CD56^dim^ NK cells after stimulation with K562 cells **(B)** or Ab-coated p815 cells **(D)**. **(C,E)** Boolean analysis of co-expression patterns of CD69, CD107a, and IFN-γ by CD56^dim^ NK cells after stimulation with K562 cells **(C)** or Ab-coated p815 cells **(E)**. Data in **(B–E)** are shown from 53 Kenyan individuals (*n* = 26 QFT^+^ and *n* = 27 QFT^−^) and 9 U.S. healthy controls. Frequencies in **(B–E)** are shown after subtraction of background expression in the unstimulated control condition. Boxes in **(B–E)** represent the median and interquartile ranges; whiskers represent the 10th and 90th percentiles. Differences among groups were assessed using a Kruskal-Wallis test, with *p*-values adjusted for multiple comparisons using Dunn's post-test. ^*^p < 0.05; ^**^p < 0.01; and ^***^p < 0.001.

Through receptor dependent mechanisms, NK cells recognize tumor and virus infected cells that downregulate MHC class I expression as an immune escape mechanism (Paul and Lal, 2017). Given the substantial differences in CD56^dim^ NK cell phenotype in the three participant groups, we next evaluated the functional capacity of CD56^dim^ NK cells to respond to the generic NK cell targets of MHC class I-devoid K562 tumor cells and Ab-coated p815 cells (Figure 3A). CD56^dim^ NK cells from U.S. healthy adults had a generally more robust response to stimulation with K562 cells, as measured by CD69, CD107a, and IFN-γ expression (Figure 3B), compared with Kenyan adults. In addition, Boolean analysis of all three markers indicated increased co-expression of two or three markers by CD56^dim^ NK cells from U.S. healthy controls, compared with Kenyan adults (Figure 3C). Interestingly, among Kenyan adults, CD56^dim^ NK cells from QFT^+^ individuals with LTBI co-expressed CD69 and CD107a at higher levels following stimulation with K562 cells, compared with QFT^−^ individuals, although this did not maintain statistical significance after correction for multiple comparisons (Figure 3C). No significant differences in CD69, CD107a, or IFN-γ expression were observed by CD56^dim^ NK cells from the three groups following stimulation with Ab-coated p815 target cells (Figures 3D,E). Taken together, these data indicate that a high pathogen burden environment in Kenya is associated with reduced NK cell reactivity to MHC class I-devoid cells, whereas Ab-mediated activation of NK cells is maintained at similar levels to those seen by NK cells from U.S. healthy controls.

### Differential Reactivity of CD56^dim^ NK Cells to Mtb Antigen Stimulation in Kenyan and U.S. Adults

**Figure 4:**
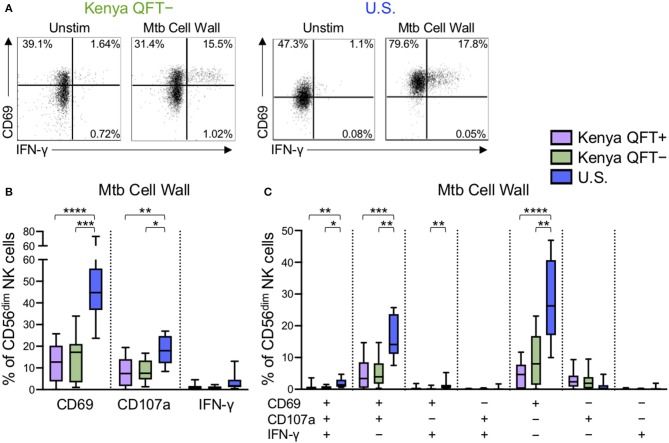
Differential reactivity of CD56^dim^ NK cells to Mtb antigen stimulation in Kenyan adults and U.S. healthy controls. PBMCs were stimulated with Mtb cell wall antigen for 24 h in the presence of 100 U/ml IL-2; PBMCs incubated with IL-2 alone, in the absence of Mtb antigen, served as a negative control (Unstim). **(A)** Representative flow plots of CD69 and IFNγ expression by CD56^dim^ NK cells from a QFT^−^ Kenyan donor and a U.S. healthy donor. **(B)** Expression of CD69, CD107a, and IFN-γ measured on CD56^dim^ NK cells after stimulation with Mtb cell wall antigen. **(C)** Boolean analysis of co-expression patterns of CD69, CD107a, and IFN-γ expression by CD56^dim^ NK cells after stimulation of PBMCs with Mtb cell wall. Data in **(B,C)** are shown from 51 Kenyan donors (*n* = 26 QFT^+^, *n* = 25 QFT^−^) and 9 U.S. healthy controls. Frequencies in **(B,C)** are shown after subtraction of background expression by CD56^dim^ NK cells in the presence of media with IL-2 alone. Boxes in **(B,C)** represent the median and interquartile ranges; whiskers represent the 10th and 90th percentiles. Differences among groups were assessed using a Kruskal-Wallis test, with *p*-values adjusted for multiple comparisons using Dunn's post-test. ^*^p < 0.05; ^**^p < 0.01; ^***^p < 0.001; and ^****^p < 0.0001.

The above data indicated that NK cell phenotype and functional responses to generic stimuli are impacted by Mtb infection status, as well as high (Kenya) vs. low (U.S.) pathogen burden settings. We next sought to determine if Mtb infection modifies NK cell reactivity to Mtb antigen stimulation. Thus, we stimulated PBMCs from each of the participant groups with Mtb cell wall antigen for 24 h in the presence of IL-2, followed by flow cytometry for expression of CD69, CD107a, IFN-γ, TNF-α, and IL-22 (Figure 4A and data not shown). CD56^dim^ NK cells expressed very low levels of TNF-α and IL-22 following stimulation of PBMCs with Mtb cell wall antigens (median <0.1% of CD56^dim^ NK cells in each group; data not shown). However, Mtb antigen stimulation induced upregulation of CD69 and IFN-γ expression by CD56^dim^ NK cells, as well as degranulation, as measured by surface expression of CD107a. Of note, there were marked differences in CD56^dim^ NK cell reactivity to Mtb antigens in healthy adults from the U.S. vs. Kenya, with U.S. healthy controls expressing significantly higher levels of CD69 and CD107a following stimulation with Mtb antigens, compared with QFT^+^ and QFT^−^ individuals from Kenya (Figure 4B).

We next evaluated Mtb cell wall-induced co-expression of CD69, CD107a and IFN-γ by CD56^dim^ NK cells. The frequency of CD56^dim^ NK cells expressing CD69, either alone or in combination with CD107a and/or IFN-γ, was consistently higher in U.S. healthy controls, compared with Kenyan adults (Figure 4C). These data suggest a heightened level of Mtb antigen-induced activation, as measured by upregulation of CD69, by NK cells from Mtb-naïve, healthy adults in the U.S., compared with Kenyan adults in a TB-endemic environment. Interestingly, among Kenyan adults, the frequency of Mtb antigen-induced CD69 single-positive CD56^dim^ NK cells was higher in QFT^−^ individuals, compared with QFT^+^ individuals, although this difference did not remain statistically significant following correction for multiple comparisons (Figure 4C).

To determine if our results of NK cell reactivity to Mtb cell wall were reproducible with other Mtb antigen preparations, we also stimulated PBMCs with Mtb cell membrane and Mtb whole cell lysate. Similar to stimulation with Mtb cell wall, stimulation of PBMCs with Mtb cell membrane and whole cell lysate antigens induced higher expression of CD69 and CD107a by CD56^dim^ NK cells from Mtb-naïve U.S. healthy adults, compared with Kenyan adults (Figure S2). Similar to our findings with Mtb cell wall, among Kenyan adults, the frequency of Mtb cell membrane induced CD56^dim^ NK cells co-expressing CD69 and CD107a was higher in QFT^−^ individuals, compared with QFT^+^ individuals (Figure S2D), although this did not maintain statistical significance following correction for multiple comparisons. Overall, these data indicate that CD56^dim^ NK cell responses to Mtb antigens are characterized predominately by upregulation of CD69 and CD107a expression, and that CD56^dim^ NK cells from Mtb-naïve adults express significantly higher frequencies of these markers, compared with Kenyan adults. Moreover, these data suggest that the capacity to restimulate NK cells with Mtb antigens *in vitro* may be further dampened in individuals with LTBI, compared with QFT^−^ individuals from the same TB endemic environment.

### *Ex vivo* Phenotype of CD56^dim^ NK Cells Correlates With Functional Reactivity to Mtb Antigens

**Figure 5:**
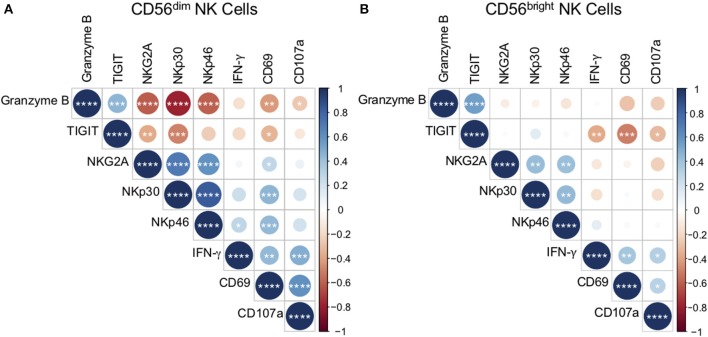
*Ex vivo* phenotype of CD56^dim^ NK cells correlates with reactivity to Mtb cell wall. The phenotype of CD56^dim^ and CD56^bright^ NK cells in PBMCs *ex vivo* was determined as described in Figure 1. Expression of CD69, CD107a, and IFN-γ by CD56^dim^ and CD56^bright^ NK cells following stimulation with Mtb cell wall was determined as described in Figure 4. Correlations between CD56^dim^ **(A)** and CD56^bright^ **(B)** NK cell phenotype and Mtb cell wall-induced effector function were evaluated using a non-parametric Spearman rank correlation. Correlograms were generated using the corrplot function in R. Positive correlations are displayed in blue and negative correlations in red. Color intensity and the size of the circle are proportional to the correlation coefficients. Phenotypic markers and effector molecules within the CD56^dim^ plot were ordered using the Ward method of hierarchical clustering. The order was then applied to the CD56^bright^ data. ^*^p < 0.05; ^**^p < 0.01; ^***^p < 0.001;and ^****^p < 0.0001.

NK cells can directly recognize pathogens through antigen interaction with NK cell receptors (Esin et al., 2008; Li et al., 2013). To better define the relationship between NK cell receptor expression *ex vivo* and functional responses to Mtb antigens, we generated a correlation matrix of CD56^dim^ NK cell receptor expression *ex vivo* and the frequency of CD56^dim^ NK cells expressing CD69, CD107a, and IFN-γ following stimulation with Mtb cell wall *in vitro* (Figure 5). We focused our analysis of phenotypic markers to those markers that were expressed at significantly different levels among the participant groups (see Figure 2A): NKG2A, NKp30, NKp46, granzyme B, and TIGIT. Among the phenotypic receptors, there was a strong positive correlation between expression of granzyme B and TIGIT, and between NKG2A, NKp30, and NKp46. There were also strong inverse correlations between granzyme B and NKG2A, NKp30, and NKp46 (Figure 5A). By contrast, analysis of CD56^bright^ NK cells by a similar correlation matrix approach revealed no relationship between granzyme B expression and NKG2A, NKp30, and NKp46 on CD56^bright^ cells (Figure 5B).

As expected, the frequencies of Mtb-induced NK cells expressing CD69, CD107a, and IFN-γ correlated positively with each other (Figures 5A,B). The frequency of CD69^+^ CD56^dim^ cells following stimulation with Mtb antigen correlated positively with *ex vivo* frequencies of NKG2A, NKp30, and NKp46, and inversely with *ex vivo* frequencies of granzyme B and TIGIT (Figure 5A). Furthermore, Mtb-induced expression of CD107a by CD56^dim^ cells correlated inversely with *ex vivo* granzyme B expression, while Mtb-induced induction of IFN-γ correlated positively with NKp46 (Figure 5A). No significant correlations were found between *ex vivo* NK cell expression of CD57, KIRs, or granulysin and the NK cell responses to Mtb antigens *in vitro* (data not shown).

By contrast with CD56^dim^ NK cells, significant correlations between *ex vivo* CD56^bright^ phenotype and Mtb antigen-induced CD69, CD107a, and IFN-γ expression were limited to TIGIT, with *ex vivo* expression of TIGIT correlating inversely with all three effector molecules by CD56^bright^ cells following Mtb antigen stimulation (Figure 5B). Unlike CD56^dim^ cells, there were no positive correlations between *ex vivo* receptor expression by CD56^bright^ NK cells and CD56^bright^ reactivity to Mtb antigen stimulation. Taken together, these data identify NK cell phenotypic markers that correlate with functional NK cell responses to Mtb antigens *in vitro*. In addition, these results highlight the differences in reactivity by CD56^bright^ and CD56^dim^ NK cell subsets to Mtb antigen stimulation.

### NK Cell Reactivity to Mtb Antigens Is Partially Dependent on IL-12 and IL-18

**Figure 6:**
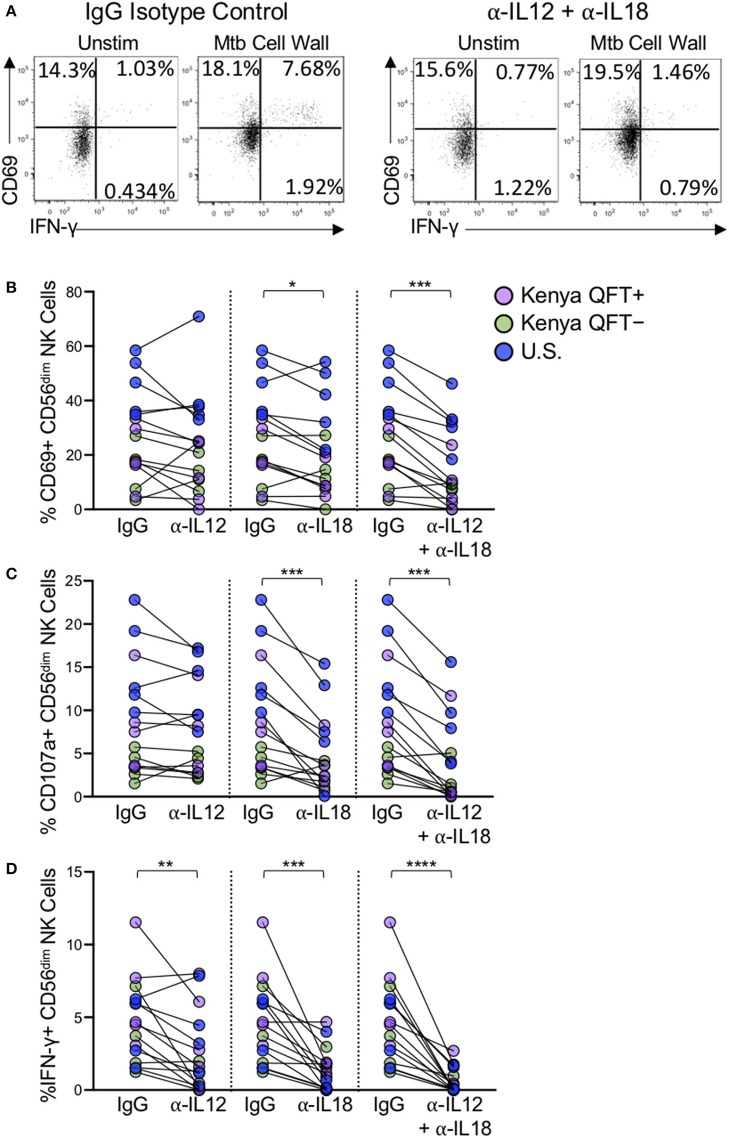
NK cell reactivity to Mtb antigen stimulation is partially dependent on IL-12 and IL-18. PBMCs were stimulated with Mtb cell wall, as described in Figure 4, in the presence of anti-human IL-12 Ab, anti-human IL-18 Ab, anti-IL-12, and anti-IL-18 Abs together, or an IgG1 isotype control. **(A)** Representative flow plots of PBMCs from a Kenya QFT^+^ individual stimulated with Mtb cell wall in the presence of either anti-IL-12 + anti-IL-18 Abs or an IgG1 isotype control. **(B–D)** CD69, CD107a, and IFN-γ expression, respectively, by CD56^dim^ NK cells in PBMCs stimulated with Mtb cell wall in the presence of anti-IL12, anti-IL-18, or anti-IL-12 + anti-IL-18 together. Data from 15 individuals are shown in **(B–D)** (*n* = 5 Kenya QFT^+^, *n* = 5 Kenya QFT^−^, *n* = 5 U.S. healthy controls). Frequencies of Mtb cell wall-stimulated CD56^dim^ NK cells in **(B–D)** are shown after subtraction of background expression in the unstimulated negative control condition. Differences between the IgG1 isotype control and anti-IL-12/IL-18 neutralizing Abs were determined using a Wilcoxon matched-paired signed rank test. ^*^p < 0.05; ^**^p < 0.01; ^***^p < 0.001; and ^****^p < 0.0001.

NK cells express a number of cytokine receptors that allow them to become activated in proinflammatory environments. It has been well-described that NK cells become activated through signaling mediated by IL-12 and IL-18 produced by activated monocytes and dendritic cells (DCs) (Fehniger et al., 1999; Son et al., 2001; Ferlazzo et al., 2004; Chaix et al., 2008; Chijioke and Munz, 2013; Leong et al., 2014). To better define the mechanism of NK cell activation by Mtb antigens, we stimulated PBMCs from each participant group with Mtb antigens in the presence of neutralizing Abs to either IL-12, IL-18, a combination of IL-12 and IL-18 together, or an isotype control (Figure 6A). Neutralization of IL-12 alone did not significantly impact Mtb-induced expression of CD69 or CD107a (Figures 6B,C), although Mtb-induced expression of IFN-γ was significantly reduced by IL-12 neutralization (Figure 6D). Neutralization of IL-18 alone resulted in a significant reduction in the frequencies of CD56^dim^ NK cells expressing CD69, CD107a, and IFN-γ following Mtb antigen stimulation (Figures 6B–D). CD56^dim^ NK cell responses to Mtb antigens were further diminished by simultaneous neutralization of IL-12 and IL-18 together (Figures 6B–D). Overall, these data suggest that CD56^dim^ NK cell reactivity to Mtb antigens, regardless of Mtb exposure or infection status, is at least partially mediated by indirect mechanisms in which Mtb antigens induce cytokine production by other cells present within PBMCs, which in turn induce expression of CD69, CD107a and IFN-γ by NK cells.

## Discussion

In this study we evaluated the phenotypic and functional profiles of NK cells from QFT^+^ and QFT^−^ adults residing in a TB-endemic region in western Kenya, and compared those with NK cell profiles in Mtb-naïve, healthy adults from the U.S. We demonstrated that CD56^dim^ NK cells from Kenyan adults have distinct phenotypic profiles and attenuated responses following stimulation with MHC class I-devoid target cells and Mtb antigens, compared with U.S. healthy adult controls. Furthermore, within Kenyan adults, we found evidence of significant downregulation of NKp46 and TIGIT expression on CD56^dim^ cells from QFT^+^ individuals with LTBI, compared with QFT^−^ individuals. Moreover, we demonstrated that NK cell responses to Mtb antigen stimulation are inversely associated with *ex vivo* expression of granzyme B and TIGIT. We further characterized NK cell reactivity to Mtb antigen stimulation and determined that NK cell reactivity to Mtb is dependent, at least in part, on IL-12 and IL-18.

NK cells in the blood of humans can be divided into three distinct populations based on expression of CD56 (Cooper et al., 2001). While CD56^dim^ NK cells are highly cytolytic and CD56^bright^ cells secrete large amounts of cytokines, CD56^neg^ NK cells have diminished cytolytic activity and cytokine production capacity (Bjorkstrom et al., 2010). Previous studies have indicated that CD56^neg^ NK cells are expanded in individuals with chronic viral infections such as HIV and hepatitis C virus (Hu et al., 1995; Mavilio et al., 2005; Gonzalez et al., 2008, 2009). Despite similar frequencies of total NK cells within the lymphocyte population, we found that QFT^+^ and QFT^−^ Kenyan adults have increased proportions of CD56^neg^ cells within the total NK cell population, compared with U.S. healthy adult controls. The increased proportion of CD56^neg^ NK cells observed in adults from Kenya, a high pathogen burden setting that is endemic for malaria, helminths, and TB (Odiere et al., 2011; WHO Publication, 2018a,b), could be an indication of NK cell activation resulting from an accumulation of pathogen exposures over time.

NK cell activity is regulated through expression of various activating and inhibitory receptors (Bryceson et al., 2006), the varied expression of which generates heterogenous populations of NK cells with high diversity (Horowitz et al., 2013; Strauss-Albee et al., 2014). The most compelling evidence for pathogen-induced differentiation of NK cells with unique phenotypic and functional properties has come from studies of CMV infection (Paust et al., 2017). The prevalence of HCMV infection in healthy Kenyan adults approaches 100% (Njeru et al., 2009), thus it was not feasible to evaluate NK cell profiles in our Kenyan groups according to HCMV status. Given the near universal prevalence of HCMV infection in Kenyan adults, we selectively enrolled HCVM^+^ U.S. adults as controls, to minimize potential differences in NK cell profiles between groups that may be attributed to HCMV infection. Thus, although all study participants were HCMV^+^, we still observed marked differences in NK cell phenotype between Kenyan and U.S. adults, particularly in the CD56^dim^ subset. CD57, a marker of differentiation on NK cells (Lopez-Verges et al., 2010), was expressed at higher levels on CD56^dim^ cells from Kenyan adults, compared with U.S. adults, thus suggesting an increased level of NK cell differentiation in adults residing in a TB-endemic region in Kenya. Expression of CD57 and NKG2C has been proposed as a marker of adaptive NK cells (Lopez-Verges et al., 2011). Unfortunately, we did not evaluate expression of NKG2C in our study participants, thus we are unable to determine if NKG2C^+^ cells are also expanded within this CD57^+^ subset of CD56^dim^ NK cells in Mtb-infected and exposed Kenyan adults.

We also observed marked downregulation of NKp30 and NKp46 expression by CD56^dim^ NK cells in QFT^+^ and QFT^−^ Kenyan adults, compared with U.S. adults. Downregulation of both NKp30 and NKp46 has been described in HCMV infection (Guma et al., 2004), yet here, among our HCMV^+^ participant groups, we observed progressive downregulation of NKp46 from the highest expression levels in Mtb-naïve U.S. adults, to intermediate levels in QFT^−^ Kenyan adults and finally the lowest NKp46 expression in QFT^+^ Kenyan adults. In addition, we observed significant downregulation of NKp46 expression on CD56^bright^ cells in QFT^+^ individuals with LTBI, compared with QFT^−^ Kenyan adults, thus providing further evidence of Mtb infection-associated changes in NKp46 expression across NK cell subsets. A previous study also reported significant downregulation of NKp30 and NKp46 expression by NK cells from patients with active TB disease, compared with Mtb-uninfected healthy adults (Bozzano et al., 2009). NKp46 has been implicated as a receptor mediating NK cell lysis of Mtb-infected monocytes (Vankayalapati et al., 2002, 2005; Garg et al., 2006), thus downregulation of NKp46 could lead to impaired NK cell lysis of Mtb-infected cells. Although we did not evaluate NK cells from patients with active TB disease in the present study, future studies directly comparing NKp46 expression levels across a spectrum of Mtb exposure, infection and disease are warranted to further define the relationship between NKp46 expression and control of Mtb.

In addition to NKp30 and NKp46, significant differences in expression of NKG2A, granzyme B, and TIGIT were also observed by CD56^dim^ NK cells from Kenyan adults, compared with U.S. adults. Among Kenyan adults, TIGIT expression was downregulated on CD56^dim^ cells from QFT^+^ individuals, compared with QFT^−^ individuals. Although no previously published studies have comprehensively evaluated TIGIT on NK cells in the setting of human Mtb infection, increased TIGIT expression on NK cells has been reported in studies of HIV-infected individuals, compared with HIV-uninfected individuals (Yin et al., 2018; Motamedi et al., 2019; Vendrame et al., 2020), indicating that TIGIT expression on NK cells may be modulated in the setting of chronic infection. Moreover, the level of expression of TIGIT on human NK cells has been associated with NK cell functional heterogeneity in healthy adults (Wang et al., 2015), and blockade of TIGIT has been reported to increase NK cell effector functions (Stanietsky et al., 2009; Wang et al., 2015; Zhang et al., 2018), thus providing rationale for exploring TIGIT as a potential immunotherapeutic target. Future studies will be required to more clearly define the role of TIGIT expression on NK cells in human Mtb infection.

Our analysis of NK cell functional capacity revealed dampened IFN-γ production to K562 tumor cells in QFT^+^ and QFT^−^ Kenyan adults, compared with U.S. healthy adults. These results are consistent with the functional profile of adaptive NK cells, which have been reported to exhibit diminished IFN-γ production to tumor cells, compared with conventional NK cells (Hwang et al., 2012; Beziat et al., 2013). By contrast with dampened responses to tumor cells, adaptive NK cells have been reported to have enhanced Ab-dependent responses, compared with conventional NK cells (Lee et al., 2015; Schlums et al., 2015). We measured Ab-dependent NK cell responses in our participant groups using Ab-coated p815 cells and did not find statistically significant differences in CD56^dim^ NK cell responses, as measured by expression of CD69, CD107a, and IFN-γ. Using a cytotoxicity assay, a previous study of adolescents in South Africa indicated enhanced NK cell killing of Ab-coated p815 cells in QFT^+^ adolescents, compared with QFT^−^ adolescents (Roy Chowdhury et al., 2018). Although we observed a trend of higher frequencies of CD69^+^CD107a^+^ NK cells following stimulation with Ab-coated p815 cells in QFT^+^ adults, compared with QFT^−^ Kenyan adults and Mtb-naïve controls, this difference did not reach statistical significance. We did not measure actual killing of Ab-coated target cells in our study and it is currently unclear whether or not QFT^+^ Kenyan adults have enhanced Ab-dependent cellular cytotoxicity (ADCC) responses, compared with QFT^−^ Kenyan adults.

Although adoptive transfer studies in mouse cytomegalovirus (MCMV) infection have demonstrated the capacity of adaptive NK cells to mediate protective immunity against viral challenge (Sun et al., 2009), definitive evidence for Ag-specific adaptive NK cells in humans has been challenging. A previous study in South Africa reported that BCG revaccination of individuals with LTBI boosts BCG-reactive NK cell responses after revaccination (Suliman et al., 2016). We initially hypothesized that QFT^+^ adults with LTBI would display enhanced NK cell reactivity to Mtb antigen stimulation, compared with QFT^−^ and Mtb-naïve adults. Contrary to our initial hypothesis, we observed the lowest reactivity to Mtb antigen stimulation by NK cells from QFT^+^ Kenyan adults, a result consistent across multiple Mtb antigen preparations (cell wall, whole cell lysate, and cell membrane). These data suggest that NK cells from QFT^+^ individuals with LTBI may either be more tolerized to Mtb or have a higher threshold by which they become activated.

A previous study of healthy adults in the U.K. reported that heterogeneity in NK cell responses to BCG was associated with KIR haplotype (Portevin et al., 2012), thus suggesting a potential contribution of host genetics to NK cell reactivity to mycobacteria. Our analysis of KIR expression was limited to CD158 (KIR2DL1/S1/S3/S5), CD158b (KIR2DL2/L3), and CD158e1 (KIR3DL1); furthermore, we did not perform KIR genotyping of the participants in our cohort, thus the potential contribution of KIR haplotype to NK cell responses to Mtb in our cohorts remains uncertain.

To better understand the relationship between NK cell phenotype *ex vivo* and functional reactivity to Mtb antigens, we performed a correlation matrix analysis and found that CD56^dim^ NK cell activation to Mtb antigens, as measured by CD69 upregulation, is inversely correlated with *ex vivo* expression of granzyme B and TIGIT, and positively correlated with expression of NKG2A, NKp30, and NKp46. These data suggest that elevated CD56^dim^ expression of granzyme B and TIGIT *ex vivo* could be indicative of attenuated NK cell responses to mycobacteria. Interestingly, the only receptor that was found to correlate positively with Mtb antigen-induced NK cell IFN-γ production was NKp46, which is significantly downregulated on both CD56^dim^ and CD56^bright^ subsets in QFT^+^ individuals. Of note, only NKp46 expression by CD56^dim^ cells, and not CD56^bright^ cells, correlated positively with IFN-γ production following stimulation with Mtb antigen. Downregulation of NKp46 by NK cells in QFT^+^ individuals with LTBI could be a potential mechanism contributing to dampened reactivity of NK cells to Mtb antigen stimulation, although it remains to be determined what role NKp46 may play in mediating NK cell activation to Mtb.

The positive correlation between NK cell reactivity to Mtb antigen stimulation and *ex vivo* expression of NKG2A, NKp30 and NKp46 by CD56^dim^ cells, and not CD56^bright^ cells, could be reflective of the higher proportions of CD56^dim^ NK cells, compared with CD56^bright^ NK cells, thus facilitating more robust detection of NK cell responses to Mtb antigens due to the higher starting number of CD56^dim^ cells than CD56^bright^ cells in PBMCs. Given the distinct transcriptional programs of CD56^dim^ vs. CD56^bright^ NK cells (Collins et al., 2019), it is also likely that there are other receptors on CD56^bright^ cells that we did not evaluate in this study that may be associated with NK cell reactivity to Mtb. Future studies in which CD56^dim^ and CD56^bright^ NK cells are sorted from PBMCs prior to Mtb antigen stimulation, as well as receptor blockade studies, will help further elucidate the contribution of each NK cell subset and receptors that are directly involved in NK cell reactivity to Mtb.

The precise mechanisms by which NK cells recognize Mtb remains an open area of investigation. We evaluated NK cell responses by stimulating PBMCs with Mtb antigens in the presence of IL-2, thus both direct and indirect mechanisms could potentially contribute to NK cell activation. Monocytes are also present in PBMCs and can recognize bacterial antigens through pathogen-associated molecular pattern molecules and produce several inflammatory mediators including IL-12 and IL-18, which are potent stimulators of NK cells (Denis, 1994; Son et al., 2001; Leong et al., 2014). Our experiments with IL-12 and IL-18 neutralizing Abs indicated that the combined blockade of IL-12 and IL-18 signaling substantially inhibited NK cell IFN-γ production to Mtb antigens. NK cell degranulation, as measured by CD107a expression, and activation, as measured by CD69 upregulation, are also significantly reduced by combined IL-12 and IL-18 blockade, although not entirely abolished. These results suggest that at least part of the NK cell reactivity measured in these assays may be due to indirect mechanisms of NK cell activation whereby stimulation of PBMCs with Mtb antigens induces cytokine production by other cell populations, such as monocytes and DCs, which in turn activates NK cells. In addition, stimulation with Mtb antigens can induce cytokine production by γδ T cells and CD1-restricted T cells (Kabelitz et al., 1990, 1991; Porcelli et al., 1992; Ulrichs et al., 2003; Montamat-Sicotte et al., 2011), which could also influence NK cell activation. In addition to cytokines, direct interaction with monocytes and DCs can regulate NK cell activity (Fernandez et al., 1999; Brill et al., 2001; Gerosa et al., 2002; Schierloh et al., 2005). NK cells from QFT^+^, QFT^−^, and Mtb-naïve controls reacted in a similar manner to neutralization of IL-12 and IL-18, thus suggesting a common mechanism of NK cell activation by Mtb antigens, regardless of Mtb infection or exposure status. Due to limited cell availability, we were not able to conduct Mtb stimulation experiments on purified populations of NK cells to better define the capacity of Mtb antigens to directly activate NK cells. Further studies are warranted to stimulate purified NK cells with Mtb, or deplete distinct subsets from Mtb-stimulated PBMCs, such as monocytes, DCs or T cells, to inform the receptors and pathways by which Mtb activates NK cells.

There are several limitations to our study, including the use of flow cytometry to evaluate a discrete number of NK cell phenotypic markers. Future studies conducting transcriptional profiling by RNA sequencing of purified populations of NK cells from individuals across a spectrum of Mtb infection and disease states will be necessary to more comprehensively define NK cell signatures associated with human Mtb infection. Another limitation in this study was that we analyzed NK cells circulating in the peripheral blood, and it remains unknown whether distinct populations of NK cells with unique phenotypic and functional profiles are present in the lungs of Mtb-infected individuals. Additionally, we stimulated PBMCs with complex Mtb antigen preparations, rather than individual Mtb antigens, and it is unknown which Mtb antigens mediate direct or indirect activation of NK cells. It is also important to note that while Kenya is a TB-endemic country, it is also endemic for other infections, including malaria and helminths (Salgame et al., 2013), which are not endemic in the U.S. While our Kenyan subjects did not have concurrent malaria or helminth infection at the time of blood sample collection for our study, it is possible that they had previous malaria and/or helminth infections, which may have contributed to shaping the NK cell repertoire. Thus, markedly different pathogen burdens and environmental exposures likely contribute to intrinsic differences in NK cell profiles between Kenyan and U.S. adults. Lastly, we enrolled Mtb-naïve healthy adult controls in the U.S. who are seropositive for HCMV to more closely match the HCMV prevalence in the adult population in Kenya. However, we do not know the duration of HCMV infection in the U.S. and Kenya participant groups; it is possible that HCMV was acquired at a younger age in the Kenyan participants, compared with U.S. controls, which could potentially contribute to shaping the phenotypic and functional profiles of circulating NK cells due to longer duration of infection. Given the low prevalence of LTBI among U.S. born individuals (Mancuso et al., 2016), we were not able to compare NK cell profiles between QFT^+^ and QFT^−^ U.S. born adults, as we did among Kenyan adults. Future studies comparing NK cell profiles between QFT^+^ and QFT^−^ individuals in a low pathogen burden, non-TB endemic environment, as well as longitudinal studies of the same individual before and after Mtb infection, will be important to better define the direct contribution of Mtb infection to NK cell phenotypic and functional profiles.

In conclusion, we performed a comprehensive analysis of phenotypic and functional profiles of NK cells from QFT^+^ and QFT^−^ adults in a TB-endemic region in Kenya and found distinct phenotypes of CD56^dim^ NK cells in these individuals, compared with Mtb-naïve healthy adult controls. Furthermore, the *ex vivo* expression of specific markers by CD56^dim^ NK cells correlated with NK cell reactivity to Mtb antigen stimulation. There is growing appreciation that a subset of individuals who are highly exposed to infectious TB do not develop LTBI and remain persistently TST^−^ and/or QFT^−^ (Chapman and Dyerly, 1964; Houk et al., 1968; Muecke et al., 2006; Morrison et al., 2008), thus suggesting the innate immune response may be capable of clearing the bacteria in some individuals (Simmons et al., 2018). Our results identify specific differences in CD56^dim^ NK cell phenotype and function in QFT^+^, QFT^−^, and Mtb-naïve individuals and inform future studies aimed at defining NK cell correlates that may be protective against acquisition of Mtb infection and progression to TB disease.

## Data Availability Statement

The datasets generated for this study are available on request to the corresponding author.

## Ethics Statement

The studies involving human participants were reviewed and approved by Kenya Medical Research Institute Scientific and Ethics Review Unit and Emory University Institutional Review Board. The patients/participants provided their written informed consent to participate in this study.

## Author Contributions

CD, LH, and GA contributed conception and design of the study. LH, JK, JO, LS, and JT performed experimental work. CD, LH, SO, FO, AC, and NG contributed to execution and oversight of experimental work, participant recruitment and enrollment, and study database management. CD and LH contributed to data interpretation, statistical analyses, and drafted the manuscript. All authors approved the final manuscript.

### Conflict of Interest

The authors declare that the research was conducted in the absence of any commercial or financial relationships that could be construed as a potential conflict of interest.
