# Supplementary material for: Distinct Human NK Cell Phenotypes and Functional Responses to Mycobacterium tuberculosis in Adults From TB Endemic and Non-endemic Regions
Source: Front Cell Infect Microbiol. 2020 Mar 24;10:120. doi: 10.3389/fcimb.2020.00120 (PMC7105570; doi:10.3389/fcimb.2020.00120)
Supplement: Supplementary file 1 [file Data_Sheet_1.pdf]

Supplementary Material

Distinct human NK cell phenotypes and functional responses to *Mycobacterium tuberculosis* in adults from TB endemic and non-endemic regions

Levelle D. Harris, Jeremiah Khayumbi, Joshua Ongalo, Loren E. Sasser, Joan Tonui, Angela Campbell, Felix Hayara Odhiambo, Samuel Gurrion Ouma, Galit Alter, Neel R. Gandhi, Cheryl L. Day*

***Correspondence:** Cheryl L. Day: cday@emory.edu

1. Supplementary Figures

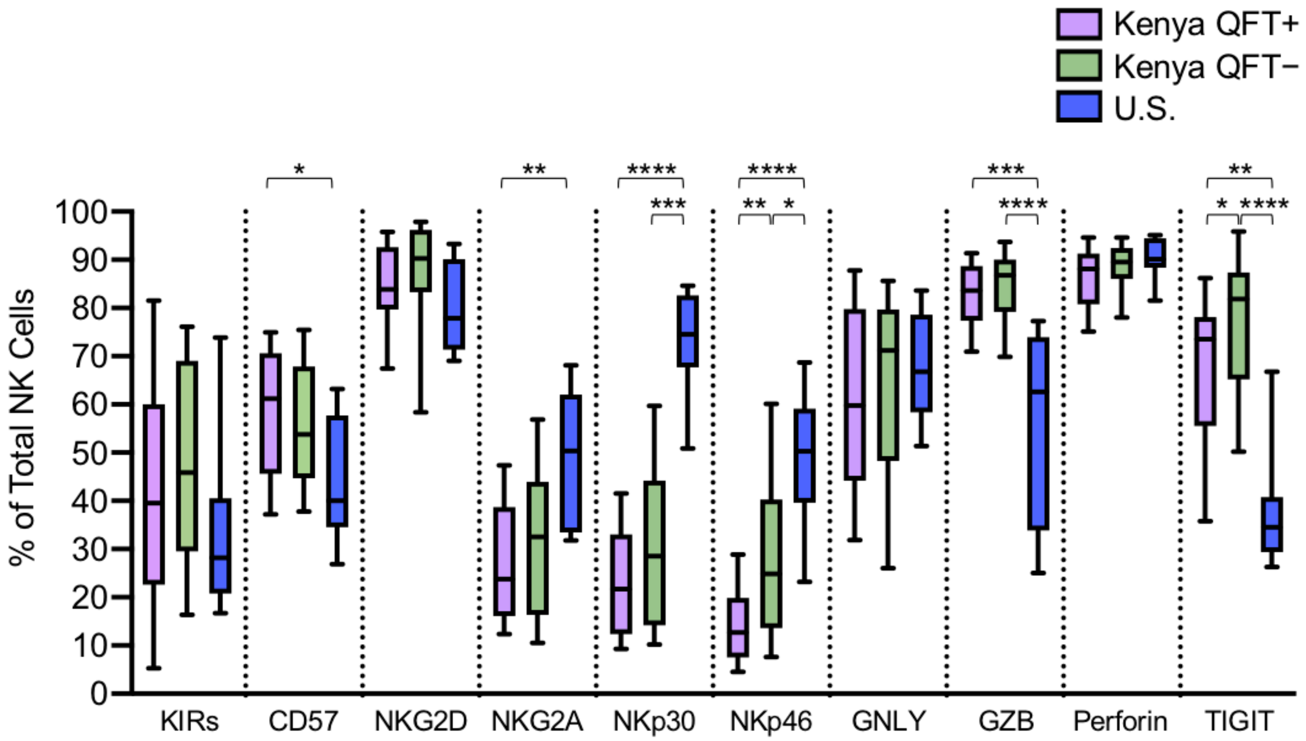

Figure S1. Phenotypic profile of total NK cells from QFT⁺ and QFT⁻ Kenyan adults is distinct from U.S. adult healthy controls. Cryopreserved PBMCs were thawed and stained for surface and intracellular phenotypic markers. Total NK cells were gated according to gating strategy described in Figure 1A. Data are shown from 30 QFT⁻ Kenyan adults, 31 LTBI Kenyan adults, and 9 U.S. healthy adult controls. Boxes represent the median and interquartile ranges; whiskers represent the 10th and 90th percentiles. GNLY, granulysin; GZB, granzyme B. Differences were assessed using a Kruskal-Wallis nonparametric one-way ANOVA, with *p*-values adjusted for multiple comparisons using Dunn's post-test. *p<0.05; **p<0.01; ***p<0.001; ****p<0.0001

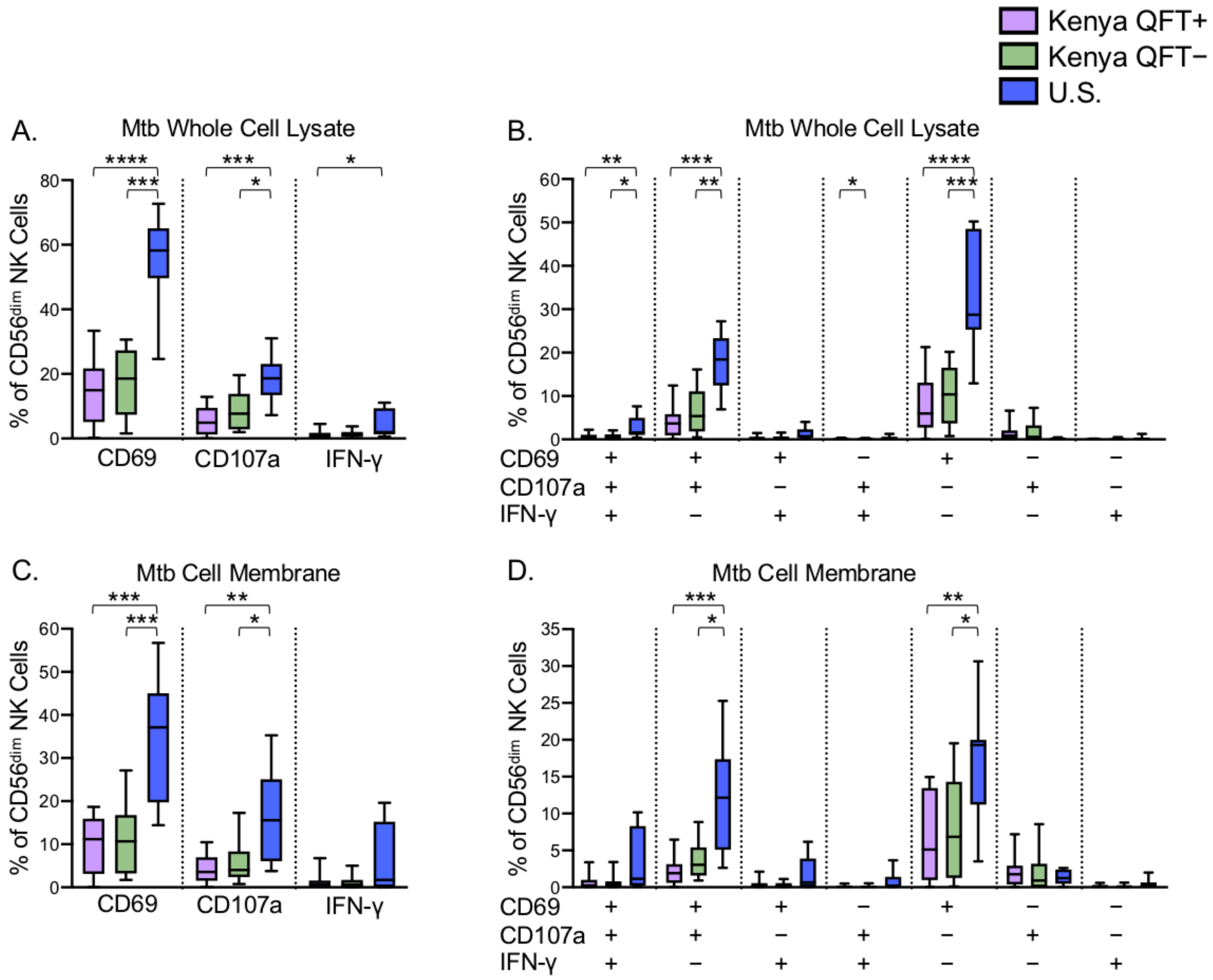

Figure S2. QFT⁺ and QFT⁻ Kenyan adults have attenuated NK cell responses to Mtb whole cell lysate and cell membrane antigens. PBMCs were stimulated with Mtb whole cell lysate and cell membrane antigens for 24 hrs in the presence of 100U/ml IL-2; PBMCs incubated with IL-2 alone, in the absence of antigen, served as a negative control. (**A**, **C**) Single marker expression of CD69, CD107a and IFN-γ measured on $CD56^{dim}$ NK cells after stimulation with Mtb whole cell lysate (A) and cell membrane (C). (**B**, **D**) Polyfunctional analysis of CD69, CD107a and IFN-γ co-expression by $CD56^{dim}$ NK cells after stimulation of PBMCs with Mtb whole cell lysate (B) and cell membrane (D). Data in panels A-D are shown from 26 QFT^{+} Kenyan adults, 25 QFT^{-} Kenyan adults, and 9 U.S. healthy adult donors. Frequencies are shown after subtraction of background expression of CD69, CD107a, and IFN-γ expression in the negative control condition (PBMCs incubated in media with IL-2 alone). Boxes represent the median and interquartile ranges; whiskers represent the 10th and 90th percentiles. Differences between groups were assessed using a Kruskal-Wallis nonparametric one-way ANOVA, with *p*-values adjusted for multiple comparisons using Dunn's post-test. *p<0.05; **p<0.01; ***p<0.001; ****p<0.0001
